# Zimbabwean secondary school Guidance and Counseling teachers teaching sexuality education in the HIV and AIDS education curriculum^*^

**DOI:** 10.1080/17290376.2019.1610485

**Published:** 2019-04-30

**Authors:** Ephias Gudyanga, Naydene de Lange, Mathabo Khau

**Affiliations:** School for Education Research and Engagement, Nelson Mandela University, Port Elizabeth, South Africa

**Keywords:** Guidance and Counseling, HIV and AIDS education, participatory visual methodology, sexuality education

## Abstract

In spite of the importance of sexuality education and HIV and AIDS education in preventing HIV infections, Zimbabwean secondary school Guidance and Counseling teachers are not engaging optimally with the current Guidance and Counseling, HIV and AIDS & Life Skills education curriculum, and hence, they are not serving the needs of the learners in the context of the HIV and AIDS pandemic. The aim of the study, therefore, was to explore how Guidance and Counseling teachers could be enabled to teach the necessary critical content in sexuality education in the HIV and AIDS education curriculum. A qualitative research design, informed by a critical paradigm, using participatory visual methodology and methods such as drawing and focus group discussion, was used with eight purposively selected Guidance and Counseling teachers from Gweru district, Zimbabwe. The study was theoretically framed by Cultural Historical Activity Theory. Guidance and Counseling teachers found themselves in a community with diverse cultural practices and beliefs of which some seemed to contradict what was supposed to be taught in the curriculum. The participatory visual methodology, however, enabled a process in which the Guidance and Counseling teachers could reflect on themselves, the context in which they taught, their sexuality education work and learn how to navigate the contradictions and tensions, and to use such contradictions as sources of learning and sources for change. The results have several implications for policy in terms of the Guidance and Counseling curriculum and engaging with cultural issues; and for practice in terms of teacher professional development, teacher training, and for stakeholder contribution.

## 1. Introduction

The first case of human immunodeficiency virus (HIV) and acquired immune deficiency syndrome (AIDS) in Zimbabwe was reported in 1985 and within two years of that reporting, the prevalence of people living with HIV in Zimbabwe among the 15–29 year age group had risen to about 29% and even further to 35% by 1992 (UNAIDS, 2008; WHO, 2007). The Zimbabwean government then initiated its first HIV and AIDS policy to guide the nation from 1987–1992, the Emergency Short Term Plan (ESTP) (Secretariat, 2011). Acting in response to this ESTP, the Government Ministries designed programmes targeting the reduction of the HIV infection rate. Furthermore, the Education Ministry together with UNICEF formulated the AIDS Action Program for implementation in all Zimbabwean schools. The programme was launched in both primary and secondary schools in November 1992 and was compulsory for all learners from grade 4 to form 6 (UNAIDS, 2008). Having noted that the name AIDS Action Program did not encompass the element of HIV and Life Skills, the Ministry adjusted the programme and changed the name to Guidance and Counseling, HIV and AIDS and Life Skills Education (MoESC, 1993) which hereafter is referred to as Guidance and Counseling (G&C). From 1993, all secondary school learners in Zimbabwe were expected to be taught Guidance and Counseling in accordance with the Chief Education Officer’s circular No. 16 of 1993 (MoESC, 1993) by Guidance and Counseling teachers.

In this article, we draw on the voices of Zimbabwean Guidance and Counseling teachers, through participatory visual research as intervention, to explain how it seemed to enable them to see how they might strengthen their teaching of sexuality education in cultural contexts where talking about sexuality is often a challenge.

## 2. Background

In this section, we start by discussing teachers’ experiences of challenges in teaching sexuality education, and then teacher professional development.

### 2.1. Teachers’ experiences of challenges to teaching sexuality education

The International Technical Guidance on Sexuality Education (UNAIDS, 2014) guides teachers on what content to teach and at what particular age in the sexuality education curricula. There is however, evidence that many countries’ curricula are not following what is expected of them (UNESCO, 2014). Consequently, teachers globally, experience challenges in teaching sexuality education (UNESCO, 2014). For instance, in Zimbabwe, sexuality education in the HIV and AIDS curriculum is taught as a stand-alone subject, whereas the International Technical Guidance on Sexuality Education recommends that the subject be infused into the other mainline subjects (UNAIDS, 2014). Therefore, this places the burden of teaching sexuality education on the shoulders of the G&C teachers alone.

The teaching of G&C is impacted negatively by several factors such as the status of G&C in a school. It is not offered as an examinable subject and it is the only subject taught once a week for about 35 min (Manzira, 2014; MoESC, 1993). Furthermore, the timetable for the Zimbabwe teacher is overloaded (Manzira, 2014; Muguwe & Gwirayi, 2011), as a result, teachers end up resorting to expository approaches rather than participatory approaches to teaching (UNESCO, 2012b). The teachers and learners also seemed not to pay much attention to it (Chifunyise, Benoy, & Mukubi, 1999). The lack of textbooks and other resources did not help matters either (Manzira, 2014; Muguwe & Gwirayi, 2011). Some trained teachers were not aware of how to teach the G&C subject (World Bank, 2002), while others reported that they were shy to teach some topics, for instance sexuality education. They argued that such topics clashed with their cultural values and beliefs, that sex was taboo to talk about, for instance, that the vernacular for human reproductive organs is taboo (Mugweni, Hartell, & Phatudi, 2013). All these issues compounded teachers’ failing to teach G&C and sexuality education within HIV and AIDS education.

Furthermore, Zimbabwe is a multicultural society made up of Shona, Ndebele, Asian, Indian and English cultures, amongst many others. The teacher does not necessarily know the cultural beliefs and values of the community from which every child comes and the how and what is taught might be seen as offensive to a particular culture. In light of this, this scenario becomes a dilemma, and, hence, a challenge to the teacher to be sensitive to the different cultures. Clearly, culture plays a key role in teaching sexuality education (Mugweni et al., 2013) and might prohibit teaching the skills to negotiate safer sex (Mugweni et al., 2013). The Zimbabwean black girl, for example, should not initiate sex, let alone safer sex, because of her cultural upbringing (Muguwe & Gwirayi, 2011). The girl grows up being taught to call her husband ‘lord’, as a way of respect, and by doing so is positioned as a minor to the husband (Manzira, 2014). Therefore, culturally, for the teacher to instil skills to negotiate for safer sex on the part of a woman, is a challenge because it would appear as if the teacher is revolting against cultural norms and values (Manzira, 2014). It will, therefore, also be difficult to assess attainment of safer sex negotiating skills (Clarke, Yankah, & Aggleton, 2015), leaving the teacher with a challenging situation.

The nature of the sexuality education and HIV and AIDS education curricula lead to several learners coming to the G&C teacher for counselling by virtue of them being affected or infected. Such counselling takes a lot of the teacher’s time and energy (Gudyanga, Moyo, & Gudyanga, 2015; Manzira, 2014). However, such work is seldom taken into consideration for promotion purposes (Pithouse-Morgan et al., 2013) and teachers may suffer burn-out as a result of caring for the traumatised learners (Pithouse-Morgan et al., 2013). In their study of South African teachers in Durban, ‘The hidden work of caring’, Bhana, Morrell, Epstein, and Moletsane (2006) argued that such care cushions both the affected and infected from different types of psychological trauma. That cushioning is not normally noticed by school authorities, although it is a big challenge and experience in the life of the teacher (Pithouse-Morgan et al., 2013).

The apparent boredom of the learners to learning G&C (Muguwe & Gwirayi, 2011) could be addressed by using participatory pedagogies, but teachers are, however, often unable to apply appropriate participatory approaches to their teaching (Bhana, 2007; Pattman & Chege, 2003). This is because they have not been sufficiently trained to use such participatory approaches, and over rely on a transmission mode to transfer knowledge (Bilinga & Mabula, 2014; Onyango, 2009), instead of engaging learners with the knowledge of sexuality education. If teaching sexuality education is to be effective, then participatory and learner centredness are key, including appropriate topic sequencing. Such approaches were found to be a challenge to teachers who may not have had such training backgrounds, let alone handling large classes whose education is examination driven (UNESCO, 2014).

However, when it comes to classroom subject learning, every learner comes into the class with some form of knowledge based on his or her experiences. Therefore, it is the teacher’s role to find appropriate means of carefully guiding the learner through the sexuality education appropriate for the developmental stage of the learner. Also, the sexuality education teacher faces challenges to guide the child through sexuality education to enable the learner to understand his or her body in relation to societal values and beliefs and the natural changes that take place within his or her body (Van der Riet, 2009; Van Rooyen & Louw, 1994). The teacher is often not skilful enough to appropriately nurture the learner, who could expect to get full assistance from the teacher (Van Laren, 2008).

### 2.2. Teacher professional development (TPD)

Teacher development is ‘the professional growth a teacher achieves as a result of gaining increased experience and examining his or her teaching systematically’ (Villegas-Reimers, 2003, p. 11). Professional development takes place as one attends conferences, workshops and seminars, gets mentored and reads professional books (UNESCO, 2015). Professional development is a deliberate, systematically planned long term process leading to the growth of a teacher and developing him or her in the profession (UNESCO, 2015). TPD entails teachers to be active constructivists of knowledge rather than mere transmitters of information (UNESCO, 2015). They are expected to engage in classroom observations, assessment in concrete tasks related to teaching, and to reflect (UNESCO, 2015; Villegas-Reimers, 2003).

Very few studies pay close attention to the teachers’ professional development in line with teaching sexuality education (Pithouse-Morgan et al., 2013). Baxen (2011) refers to the connection between teachers’ personal understanding of their role and identity as educators and how these filter or shape the critical knowledges to be taught to learners. Elements of self-reflexivity where teachers acknowledge their own personal values, beliefs and prejudices as separate from sexuality education content (Francis, 2016; Francis & DePalma, 2015), are therefore important. This however, requires time, which is often not afforded in TPD. Although these studies are from the South African researchers’ orientation, the same argument holds for Zimbabwe. From our search of the literature, we are yet to find a single Zimbabwean study on teacher self-reflection and self-reflexivity on their professional and personal growth with reference to teaching sexuality education in the HIV and AIDS education curriculum. There seems to be a knowledge gap in this area. It is also noted that policy makers know little of the role of the teacher and his/her reflexive experiences (Onyango, 2009; Wood, 2012).

Research studies identify some key issues that can be used to facilitate teacher preparation and development in their engagement with sexuality education in the HIV and AIDS education curriculum. Firstly, research has shown that interest in teaching sexuality education has a direct bearing on the ability of teaching the curriculum, hence, the teaching of sexuality education is not for every teacher (FOSE, 2015; Mitchel, De Lange, Moletsane, Stuart, & Buthelezi, 2005). It is for those teachers who are comfortable and committed to teach sexuality education who can enable the learners to effectively engage with the curriculum. Such teachers will demonstrate their awareness of their beliefs and values towards sexuality education (FOSE, 2015).

Second, is the notion that practicing teachers are expected to have biological content knowledge of human sexuality that is correct and current. If such knowledge is compromised then the teaching likewise gets compromised (FOSE, 2015; Mitchel et al., 2005). Third, teacher professional development should prepare teachers to be able to use modern communication technologies. Instead of being information gatekeepers, teachers should assist learners to make meaning of the received information. Moreover, teachers should draw on learners’ existing knowledge and see the learners as constructors and co-constructors of knowledge (Francis & DePalma, 2015; Moletsane et al., 2007). In this way the proposed four pillars of education i.e. ‘learning to know, learning to do, learning to live together and learning to be’ (UNESCO, 2014, p. 13), become more meaningful. However, today’s teacher may not be able to do so as a result of inadequate training in sexuality education (Bilinga & Mabula, 2014; Onyango, 2009).

Fourth, the length of time that teachers spent on training was noted to have a bearing on the effectiveness of the teacher who teaches sexuality education. Some correlation was noted between the duration of training and the amount of sexuality education and HIV and AIDS content taught to learners in some sub-Saharan African countries (UNESCO & UNPF, 2014). Short term training courses were noted to be inadequate to give teacher confidence, competence and positive attitudes (Onyango, 2009). Pre-service teachers who received professional training in sexuality education in HIV and AIDS education curriculum followed by further re-training as in-service teachers, seemed to gain more knowledge and skills (Kelly, 2009).

Fifth, research has also shown that one of the important issues during professional development is to enable both the pre- and in-service teachers to learn through engagement and deep thinking through creative and participatory strategies. This approach enables teachers to think deeply about their teaching. For instance, teachers will get to think about teaching sexuality education. They reflect on social issues that impact directly and or indirectly on their professional and personal development thereby often coming up with issues that matter in their profession (Bilinga & Mabula, 2014; Pithouse-Morgan et al., 2013). Creative and participatory engagement with sexuality education gives teachers room to further interrogate and introspect on their teaching and on the learner’s learning (Francis, 2010; Francis, 2011; Pithouse-Morgan et al., 2013). The approach under discussion was noted to make a positive change in the development of teacher participants. One participant in the study was cited as saying ‘I feel a lot more strongly about the topic than I thought. I also didn’t realize how it affects me as a person until now’, that is, after having gone through engagement and deep thinking about some issue through a participatory approach (Pithouse-Morgan et al., 2013, p. 86).

Sixth is the issue of dialogue and sharing. It is prudent for teachers of sexuality education in HIV and AIDS education to learn to dialogue and share their lived experiences (Msutwana & De Lange, 2017; Pithouse-Morgan et al., 2013). These researchers argue that there is value in listening as other colleagues share their lived experiences of HIV and AIDS education. It was noted that through dialoguing and sharing, through creative and participatory activities, teachers might end up perceiving issues differently, even perhaps doing it from the perspective of the teacher who will be sharing his or her story(ies). Mitchell and De Lange (2013), for example, used participatory video as method to make cellphilms (a film made with a cellphone) when they worked with in-service teachers to deepen the teachers’ understanding of HIV and AIDS education as prevention. However, when they noted faulty knowledge and perceptions which could be detrimental to the well-being of the learners, they used a ‘speaking back’ approach to get the teachers to deliberately ‘speak back’ to the content in their cellphilms, i.e. to rethink and readjust their knowledge and perceptions and portray it in their revised cellphilms.

Lastly, is the issue that teaching sexuality education, like any other school subject, should bring joy and fun to both teachers and learners (De Lange & Stuart, 2012). From their study, Pithouse-Morgan et al. (2013) argue that enjoyment can be derived from creative and participatory activities in teaching HIV and AIDS issues which could, at times, be disheartening. As participants worked through their activities through the drawings, videos and photo creations, a sense of excitement was noted. Therefore, as part of teacher professional development, there is need to develop a sense of hope and pride in one’s life through education (Olivier, Wood, & De Lange, 2009; Pithouse-Morgan et al., 2013).

It is noted that in spite of the importance of education, and HIV and AIDS education in preventing HIV infections (Bhana et al., 2006; De Lange & Stuart, 2012; Kelly, 2002; Plan-International, 2013; Wood & Hillman, 2008), Zimbabwe secondary school G&C teachers are not engaging optimally with this current G&C, HIV and AIDS and Life Skills Education curriculum, and hence, they are not serving the needs of the school children in the context of HIV and AIDS (Chifunyise et al., 1999; Mangwaya & Ndlovu, 2012; Manzira, 2014; Mufuka & Tauya, 2013; Muguwe & Gwirayi, 2011; Mugweni et al., 2013; Musengi & Shumba, 2013). The aforementioned studies have explored this phenomenon and have deepened our understanding of why the secondary school G&C teachers are not engaging with the sexuality education within the HIV and AIDS education curriculum in the Zimbabwean context. The aim of this study is to explore how G&C teachers can be enabled to teach the necessary critical content in sexuality education. The research question therefore is: How can G&C teachers be enabled to teach the necessary critical content in sexuality education in the HIV and AIDS education curriculum in Zimbabwe secondary schools?

## 3. Theoretical framework: Cultural Historical Activity Theory

In order to frame the study, we drew on the Cultural Historical Activity Theory (CHAT) which originated in the 1920s from Vygotsky (Yamagata-Lynch, 2010), in particular on Engeström (2001) who produced the third generation CHAT, centred on Activity Systems analysis. The unit of analysis is Activity Systems. An Activity System is made up of a group of people who are focusing on some goal or purpose which they want to achieve (Yamagata-Lynch, 2010). With reference to this study, the Activity System under consideration is a group of eight G&C secondary school female teachers (participants) whose main purpose was to interact in co-producing data on how they could be enabled to teach sexuality education within the HIV and AIDS education curriculum suitable for the Zimbabwe secondary school context.

## 4. Context and participants

The study was conveniently carried out in Gweru, (where one of the researchers resides), a district in the Midlands province of Zimbabwe, working with a small purposive sample (Creswell, 2014) of eight G&C secondary school female teachers (age range 36–56 years; and teaching forms 1–6) from surrounding urban schools. Gweru district, in 2013, had an HIV prevalence of 12.5% among pregnant women aged 15–24 years (AVERT, 2015). Our sample does not profess to be representative and does not enable the generalisation of results beyond the selected participants and their context (Cohen, Manion, & Morrison, 2011; Creswell, 2014), but it did enable gaining insight into the teaching of G&C.

## 5. Methodology

In this study, we chose to use a qualitative approach, which is inductive, holistic and naturalistic, to explore the research question, drawing on the participants’ lived experiences. Furthermore, we chose to locate this study within the critical paradigm which is an integration of critical research traditions and emancipatory praxis which are applied in social sciences (Willis, 2007). It aims at emancipatory action as it tries to expose contradictions in the life worlds of the participants (Brink, Van der Walt, & Van Rensburg, 2012). Researching in the critical paradigm does not seek to merely understand reality, but to change it by redressing injustices found in the field site (Taylor & Medina, 2013). Such a paradigm is suitable for our study where we work with female G&C teachers and guided us to look for an appropriate research methodology.

As the critical paradigm deals with empowerment, we therefore, chose participatory visual methodology, which could enable participants in making their voices heard (Pithouse-Morgan et al., 2013). This methodology also involves methods which encourage deeper engagement with participants (De Lange, 2012; Pithouse-Morgan et al., 2013). Among several methods applicable to participatory visual methodology, we selected drawing, requiring participants to represent their thoughts through making drawings and adding captions (Guillemin, 2004), followed by robust discussion. We also employed focus group discussions as a tool to generate data (Greeff, 2011).

### 5.1. Drawing

Drawing is a method in which participants actively represent their ideas by making drawings, thus enabling them to describe, reflect and evoke emotions as well as pay attention to things in novel ways (Guillemin, 2004). Drawing is a powerful and perceptive research tool to explore how people make sense of their world (Guillemin, 2004). It is a simple method where only paper and pen/pencil are needed. Participants draw and then write a caption – a brief explanation of the drawing next to or below the drawing. Drawing offers a powerful way of communication which words on their own often cannot. We used drawing to engage the participants in thinking about the topic under study.

### 5.2. Focus group discussion

Focus group discussion is a method in which people are able to express their feelings and their thinking about an issue in a relaxed atmosphere (Greeff, 2011). Normally, it is a small group of people who are called together to discuss an issue or a topic in order to generate data (Wong, 2008). Focus group discussion has the advantage that participants freely contribute their ideas in a non-threatening environment. The participants are selected based on the fact that they have something in common in relation to the topic, hence, their interpretations of the topic would be both deep and contextual (Greeff, 2011). Additionally, focus group discussion is used as a means to help a researcher better understand the participants’ lived experiences.

Every participant has her own understanding and interpretation of sexuality education in the HIV and AIDS education curriculum. In the focus group discussion, the participants conveyed their understandings of the phenomenon and so large amounts of data could be generated within a short period of time (Greeff, 2011). In light of the foregoing, we found focus group discussion an ideal method of data generation for the research question.

### 5.3. The research process

We asked participants, in the first session, to use pen and paper to draw what they experienced as challenges to teaching the necessary critical content in sexuality education within the HIV and AIDS education curriculum in Zimbabwe secondary schools. We provided the following prompt: Using the pen and paper provided, make a drawing that shows the challenges to teaching sexuality education.

We encouraged them not to worry about the artistic beauty of the drawing and asked them to write a caption for their drawing. We allowed them 15 min to make their drawings. Once they had completed their drawing they displayed them on the walls of the room we were working in. Each participant explained what their drawings depicted as challenges to teaching the necessary critical content in sexuality education within the HIV and AIDS education curriculum in Zimbabwe secondary schools.

After highlighting such challenges, participants in their focus group discussion reflected and critiqued what teachers experienced as challenges to teaching the necessary critical content in sexuality education within the HIV and AIDS education curriculum. We used a ‘speaking back’ approach in the focus group discussion, which encourages reflection on the part of the participants who had made the drawings (Mitchell & De Lange, 2013). ‘In a “speaking back” approach, the most appropriate people to interrogate images are those who produce them’ (Mitchell & De Lange, 2013, pp. 4–5). The G&C teachers reflected on and interrogated the meanings of the drawings they had made and which were posted on the walls of the room we were working in. They used these to leverage suggestions and solutions on how to overcome the challenges they experience in teaching sexuality education within the HIV and AIDS education curriculum. In the focus group discussion, we constantly probed and also referred them back to the sets of drawings displayed to encourage them to link their discussions to their earlier work and, therefore, to generate relevant and doable solutions. The explanation of the drawings and the focus group discussion were video and audio recorded. We kept these recordings in a laptop for transcription and further analysis.

### 5.4. Data analysis

A first layer of participatory analysis was done by the participants themselves, through writing captions and explaining what their drawings meant. We then did a thematic analysis of the drawings and captions and focus group data, followed by re-contextualising data in existing literature, and making meaning of the findings in terms of the theoretical framework.

In carrying out thematic analysis, we drew on Braun and Clarke (2006). Six steps of thematic analysis were employed, which are: immersion in the data, generating initial codes and categories, searching for sub-themes, reviewing sub-themes, defining and naming sub-themes, and finally presentation of the sub-themes and the themes (Braun & Clarke, 2006).

### 5.5. Trustworthiness and ethics

Trustworthiness was ensured by drawing on four major constructs, which are: credibility, transferability, dependability and confirmability (Lincoln & Guba, 1985). We constantly reflected and reframed our thinking to minimise our bias. Additionally, many hours were spent on data generation and analysis with the participants, which enabled deep engagement with the data (Creswell, 2014). The Nelson Mandela University ethics committee granted ethical clearance (Ethical Clearance number H16-EDU-ERE-005) to go into the field for the purpose of data generation. We were committed to working ethically with the participants from the outset of the study, also in how we represented their work. Written and signed informed consent were gained and pseudonyms were used for the purposes of anonymity and confidentiality.

## 6. Findings

In this paper, we draw on the analysis of drawings and on the focus group discussion to present the findings in response to the research question. The participants expressed how they could be enabled to teach the necessary critical content in sexuality education in the HIV and AIDS education curriculum in Zimbabwe secondary schools in three themes: creating a community context for talking about sexuality education; creating a school context for teaching sexuality education; and creating a space for teacher reflexivity and agency to teach sexuality education.

### 6.1. Creating a community context for talking about sexuality

This theme consists of two categories which are: drawing on community structures to break the taboo of speaking about healthy sexuality; and engaging with culture in relation to healthy sexuality.

#### 6.1.1. Drawing on community structures to break the taboo of speaking about healthy sexuality

The participants indicated that they needed the support of other community members like the police, community health workers and school development associations to assist them to overcome the challenges they experienced so that they could teach the necessary critical content in sexuality education in the HIV and AIDS education curriculum. Participants made several presentations:

Apart from the police, health workers and school development association can also assist us by having the same road show campaigns as a solution to the challenge on taboos. *(Sarah, 52-year old teacher)*

Furthermore, another participant had this to say:

The police as part of our community and law enforcement agents will assist us by breaking the culture of silence, since people don’t want to talk about those things [sexuality education]. *(Vongai, 42-year old teacher)*

It appeared that participants were of the opinion that campaigns could assist in changing cultural beliefs which oppose open discussion on sexuality education. If the police go onto the streets and campaign openly for healthy sexuality, then, perhaps, the culture of silence on sexuality education by both parents and teachers would be minimised. In that case, teachers might find it easier to implement their teaching of critical content in sexuality education in the HIV and AIDS education curriculum.

The participants in this study, interestingly, indicated the need to work in association with the police in breaking the silence on cultural taboos that are noted to be impacting negatively against the teaching of sexuality education. Working with the community outside the school is consistent with a study on the impact of HIV on education where it was reported that the success of teaching sexuality education also depends on engaging the community outside the school (Kelly, 2009). Therefore, this might imply engaging influential community leaders like the chiefs and political leaders. If the community shows the zeal and eagerness to work together with the teachers, then the implementation and teaching of HIV and sexuality education becomes sustainable (Steinhart et al., 2013). Such collaboration of community leaders and teachers could enable the parents and their extended families to deepen their understanding of sexuality education as they break the taboo of speaking about healthy sexuality.

There are several issues which prevent effective teaching of sexuality education of which cultural taboos is one. In this study, the participants expressed that taboos are yet to be overcome if effective teaching of sexuality education within HIV and AIDS education curriculum is to take place. Such need to break the silence on cultural taboos relating to sexuality education is not only a Zimbabwean challenge. In Latin American countries (Steinhart et al., 2013), sub-Saharan African countries (Weiler & Weiler, 2012), and Muslim society (Pohan et al., 2011) it was noted that teachers offend parents when they discuss issues in the classroom relating to sexuality education. Teachers and learners feel uncomfortable to discuss sexuality education issues where cultural taboos are common (Pohan et al., 2011). In countries in the Pacific and Asia, the issue of taboos relating to HIV and AIDS education leads to sexuality education not being taught at all (Ross, Dick, & Ferguson, 2006; UNESCO, 2007). Therefore, if cultural taboos are ‘untabooed’, teaching of sexuality education could be accomplished.

#### 6.1.2. Engaging with culture in relation to healthy sexuality

As a way of enabling the community to critically engage with culture in relation to healthy sexuality one participant said:

Then on culture, we also said people should be exposed to relevant information, so that they can make their own choice, because we talked about the Kalanga culture, and we are saying, if they know, if they have got knowledge of what is right, even if it is in their culture, they will have to make their own choice, to choose between their culture and the correct thing. *(Vongai, 42-year old teacher)*

When Vongai referred to an aspect of the Kalanga culture, (where a bride is given to the father-in-law for sex prior to her bridegroom), participants indicated that the communities are to engage with culture in relation to healthy sexuality in the context of HIV and AIDS, in order to get a clearer understanding of the implications of some cultural practices. If the parents were more knowledgeable, the participants felt that such parents could assist by spreading the knowledge of the dangers of such cultural practices. The same participant continued:

In our meetings with parents, we can ask some parents to address us on their views about sexuality education. Through such dialogue, it will also assist us as we teach sexuality education to their children. So we will teach at school, so they will teach also at home. *(Vongai, 42-year old teacher)*

Teachers are expected to interact with the parents and the local community in as far as the teaching of sexuality education is concerned (UNESCO, 2009). Some research from Malawi and Uganda demonstrated that if community members are involved in the sexuality education teaching programme, parents become more sensitised and opposition to the teaching and learning of sexuality education is reduced (UNESCO, 2009).

Teachers are part of the community and, therefore, are not invulnerable to the effect of the societal norms, religion and culture when it comes to teaching sexuality education (Corona & Arango, 2010). Therefore, there is a need to strengthen the teacher-parent relationship in discussing sexuality education issues (Plummer, 2010) in relation to culture.

### 6.2. Creating a school context for teaching sexuality education

The theme has two categories, namely strengthening the G&C teachers professionally, and leadership support.

#### 6.2.1. Strengthening the G&C teachers professionally

The participants indicated the need for strengthening the G&C teachers professionally, meaning that the teachers should be trained. In her initial presentation, one participant stated:

As G&C teachers, we are incompetent in our subject, we need training. We are supposed to be exposed to relevant information for us to be knowledgeable*. (Vongai, 42-year old teacher)*

Furthermore, another participant stated:

We are saying, the Ministry should train us or hire professionally trained teachers. We said, some teachers, you are a Geography teacher and you are just given a G&C lesson to teach and you have no knowledge in that area. So, in the end, you are going to develop a negative attitude. *(Esinia, 38-year old teacher)*

Another participant revealed that:

Remember, we are not trained to teach G&C. Our Ministry, it is its responsibility to train specialized teachers in G&C as it does with other teaching subjects. *(Edith, 56-year old teacher)*

In her presentation, another participant furthermore reiterated the need for teachers to be trained. She had this to say:

Teachers must be trained to become qualified counselors of HIV & AIDS and good teachers of sexuality education*. (Shuvai, 51-year old teacher)*

Additionally, in order to ground the teachers in their work, participants referred to the need for G&C teachers to understand the context in which they live and work and teach according to the context. One participant explained:

When we look at the Zimbabwean curriculum, the teaching of sexuality education, there are certain gaps which are there within the Zimbabwean curriculum and these gaps for example, the aspect that, certain aspects of sexuality is not really emphasized though there is a syllabus. We are saying that need to fill in that gap which exists in the Zimbabwean curriculum and in order to fill in the gap, you have to investigate on the challenges which are being faced by the teachers who are teaching sexuality education and also try to find solutions as we were doing. *(Esinia, 38-year old teacher)*

Of the eight participants, none was involved in the initial training of 1992. For those Zimbabwe secondary school G&C teachers who got the initial training at the commencement of the HIV and AIDS education programme in 1992, only two weeks were allocated to such a mammoth task (Mufuka & Tauya, 2013; World-Bank, 2002). Generally, the length of time that teachers spent on training was noted to have a bearing on the effectiveness of the teacher who teaches sexuality education (Kelly, 2009). Some relationship was noted between the duration of training and the depth of sexuality education and HIV and AIDS content taught to learners in some sub-Saharan African countries (Onyango, 2009).

The teaching of sexuality education is necessary in both the pre-service and in-service programmes in Teachers Colleges and Universities (Preston, 2013). Studies have shown that during the pre-service programmes, the future teachers have a setting and opportunity for exploring their own concerns and beliefs relating to sexuality education (Kelly, 2009; Preston, 2013; UNAIDS, 2013; Wood, 2012).

Action research-based type of teacher training enables pre-service and in-service teachers to assess their own attitudes towards and experiences of teaching sexuality education (UNESCO, 2014). It also encourages the future teachers to introspect and reflect on their own positioning, and to dialogue with colleagues about effective methods and approaches of teaching sexuality education (Onyango, 2009; Wood, 2012). Such action-based approaches are consistent with the preparation for ‘life-long learners who are able to learn from their own practice while maintaining reflective dialogue with other teachers, subject specialists and researchers’.

The participants in this study made it clear that they were not G&C specialists. They were hired to teach G&C for different reasons as suggested by their respective heads of schools. In some similar circumstances where there were no specialist teachers in sexuality education, it was noted that explicit teaching on sexuality became rare (Preston, 2013). In such a case it implied a serious gap in knowledge and skills amongst teachers and learners (Preston, 2013). This meant that the needs of the learners remained unmet. Therefore, there is need for explicit teaching on sexuality education in order to meet the needs for learners.

During the training of both in-service and pre-service teachers, one of the key issues to be considered was that the tutors are expected to be well trained and knowledgeable about sexuality education, in order to be effective (UNESCO, 2009). However, participants noted the absence of such training in Zimbabwe leaving the teachers with a superficial understanding of what and how to teach.

#### 6.2.2. Leadership support

The participants lamented about the lack of adequate support from school heads. One participant explained:

The school administration (heads) must have a positive attitude towards the teaching of G&C. There must be well timetabled sessions or slots for G&C. The school must encourage G&C teachers to teach the subject. *(Shuvai, 51 -year old teacher)*

Furthermore, another participant also remarked:

Heads of schools have a challenge, they should change their attitude, to make it positive. They should also hire or purchase or provide us teaching materials which are relevant to the teaching of sexuality education. *(Edith, 56- year old teacher)*

Additionally, another participant made the following comment:

We need school administrators who have a positive attitude towards G&C, who are able to visit hospitals collecting charts, fliers and other leaflets that we can use for teaching sexuality education. The school heads must support us. *(Shuvai, 51- year old teacher)*

Along the same line of argument, another participant went on to say:

Heads must provide teachers with more teaching materials for use in their lessons. They should provide G&C teachers with normal to above normal teaching resources*. (Nyasha - the youngest - 36 years old teacher)*

Another participant furthermore stated:

School heads, once they show a positive attitude towards G&C, if they support us, we are ready to teach the subject. Now some of our teachers are beginning to slacken because they are seeing that the heads are not caring whether G&C is on the timetable or not. It is the school heads, who should even lobby for G&C to be examined, hence, everybody will be on his or her toes. *(Sarah, 52-year old teacher)*

It is acknowledged that not every teacher can be called upon to engage with sexuality education (Kelly, 2009; Onyango, 2009; Weiler & Weiler, 2012; Wood, 2009a). It is key to know what to teach and how to teach it but most importantly, a reassuring policy from the Ministry supporting sexuality education teachers is necessary (Weiler & Weiler, 2012). The participants in this study, who are teachers of sexuality education in the HIV and AIDS education curriculum, expressed a lack of such support and assurance. They neither got it from their line Ministry nor from the local school heads. In the International Technical Guidance on Sexuality Education report, it is argued that the support from school administration and school heads was noted to serve as a motivating tool for the sexuality education teachers (UNESCO, 2009).

Although it is argued that sexuality education teaching materials may not be very expensive (Petersen, de Beer, & Dunbar-Krige, 2011), the participants in this study expressed the need for their school heads to secure more resources for them to be able to effectively teach sexuality education within the HIV and AIDS education curriculum. In Timor-Leste, inadequate resources were noted to be a barrier to the teaching of sexuality education and training (UNESCO, 2012a). This is consistent with the argument expressed by the participants in this study, where school leadership is not supportive.

We further argue that, with plenty or few teaching resources, or with minimum support from school heads, it is still the responsibility of the teacher to be conscious of the nature of their work - a call to a labour of love and caring (Bhana et al., 2006; Pithouse-Morgan et al., 2013). Consequently, teachers are to be innovative, resourceful, original, and encouraged to think creatively of possible approaches to employ to achieve the goals as stated in the International Technical Guidance on sexuality education report (UNESCO, 2009).

### 6.3. Creating a space for teacher reflexivity and agency to teach sexuality education

The theme has five categories, namely, sensitised towards cultural diversity, how to talk about sexuality, to teach and not to preach, and seeing possibilities of a new participatory method of teaching and, together becoming ‘overcomers’.

#### 6.3.1. Sensitised towards cultural diversity

The participants focused on how the methodology enabled them to participate, reflect and think about what is holding them back from optimally teaching sexuality education, as well as on their agency to teach the necessary critical content. They came up with their own solutions for some of the challenges.

The participants’ data revealed their realisation of the diversity in terms of culture and tradition in class. One of the participants remarked that:

I learnt that so many factors impede the teaching of G&C including, language, tradition and culture. *(Esinia, 38 years old)*

In addition, another participant had the following experience to share:

I learnt a lot about challenges to teaching sexuality education, including how to teach in different cultures and religions. *(Vongai, 42- year old teacher)*

Vongai became conscious that she could teach sexuality education to learners from different cultural backgrounds. She went on to say:

I now know that children have different cultural expectations*. (Vongai, 42-year old teacher)*

This seemed to be a new awareness as her statement shows that she had reflected and thought of how she could teach the critical content in sexuality education.

Another participant exclaimed:

I learnt that, where I don’t know the culture of my children, I should ask. *(Nyasha, 36 years old teacher)*

The remarks by Nyasha showed how she had become sensitive towards the fact that learners had different cultural backgrounds, which she had to get to know about and then take cognizance of when she taught them. At this point, she was conscious that inadequate knowledge of the cultural backgrounds of her learners was holding her back from optimally teaching sexuality education but also of her agency to teach the necessary critical content in HIV and AIDS education with more awareness of the cultural diversity in her class.

Another gave a warning to herself and others when she stated:

We must be very careful when we teach pupils in boarding schools who come all over. *(Rose, 51-year old teacher)*

This is important in the context of Zimbabwe boarding schools enrolling learners from all corners of the SADC region. Such seemingly new awareness and sensitivity shows that the participatory visual methodology used in this study enabled the G&C teachers to reflect and think on how they could teach sexuality education within HIV and AIDS education curriculum.

This study seemed to enable the participants to critically look at how they could teach in a culturally sensitive way. It is argued that knowledge of the cultural backgrounds of the learners influences the success of teaching sexuality education (Bearinger et al., 2007). Rutgers (2017) asserts that for any impactful implementation of comprehensive sexuality education, it is important to engage with and involve the wider community to reduce contradictions which might arise from a diversity of cultural practices. The learners they teach live in communities and acquire the expected attitudes, values and norms, which should be mediated in class. It is the realisation by teachers of the diversity in terms of culture and tradition in class, which influences the level at which learners will engage with the learning material. For example, it is asserted that most sexuality education fails as a result of placing emphasis on abstinence, being faithful and condom usage, without aligning that ABC with the socio-cultural context of the learners (Haberland & Rogow, 2015). Studies have shown limited behaviour changes among young people as a result of such misalignment (Berglas, Constantine, & Ozer, 2014; Gallant & Maticka-Tyndale, 2004). Therefore, participants in this study recognised that for them to be enabled to teach the critical content in sexuality education within the HIV and AIDS education curriculum, understanding the cultural setting in which they teach is important.

#### 6.3.2. How to talk about sexuality

This category focuses on the G&C teachers realising the dilemma of speaking about sexuality and what language and concepts to use. Participants have, however, come up with their own plans to get around the challenge. The skill of creating solutions to the challenges at hand, demonstrates agency and resonates well with the critical paradigm, which guides this study.

One participant explained:

There are so many restrictions when teaching, like language barrier. *(Rose, 51- year old teacher)*

The statement was reaffirmed:

The first one is language as taboo, therefore, I must be careful on what to say. *(Esther, 56-year old teacher)*

Furthermore, another participant remarked:

Then on taboos, we also say people must be very sensitive with what they say. *(Shuvai, 51- year old teacher)*

The participants reflected and thought deeply about talking about sexuality in the class and had discussed the limitations of some Shona vernacular language. The names for male and female reproductive organs are taboo, as explained earlier on, however one of the participants expressed:

I shall create or coin my own new words in Shona like nhengo, to avoid being called an immoral teacher. *(Edith, 56-year old teacher)*

Although Edith had shown reflexivity, awareness (of the need for respectable language), and agency, perhaps coining of new words of her own which might not be fully recognised by learners is not a solution, rather, if she was to learn all the respected terms for sexuality within the context of Zimbabwe, then it would be helpful.

The participant went on to argue that:

Some pupils feel like expressing themselves in vernacular whilst the teacher feels comfortable to explain in English. *(Edith, 56-year old teacher)*

One of the participants reiterated:

As a G&C teacher, I should find the suitable language for me with the pupils if they are to trust me. *(Nyasha, 36-year old teacher)*

She was aware of the dilemma of how to speak about sexuality and thought that she could apply her mind in resolving the issue.

Teachers who teach sexuality education often point out that one of the most challenging aspects of teaching sexuality is handling the interaction between themselves and the learners - through communication in general, but also specifically with regard to the language related to sexuality (Helleve, Flisher, Onya, Mukoma, & Klepp, 2011). The G&C teachers concurred on the use of colloquial vocabulary, even switching between languages, as well as creating inoffensive terms. This is consistent with the UNESCO’s International Technical Guidance on Sexuality Education Report which asserts the importance of a clear and specific sexuality vocabulary (UNESCO, 2009). The Shona culture is not an exception in respecting not calling out names of reproductive organs in public. The UNESCO (2009) report says, ‘all cultures have different ways of respecting privacy and bodily integrity’ (p. 25) and stipulates the importance of accurate language for sexuality education. The participants in this study seemed to have come to that realisation through their participation in this study.

#### 6.3.3. To teach and not to preach

The participants demonstrated an awareness of the importance of teaching sexuality education. One participant stated that:

Though time might be limited to overcome the challenges pointed out, I shall keep on teaching the subject. Regardless of age, religion and culture, the aim is to focus on imparting knowledge*. (Esinia, 38- year old teacher)*

Furthermore, another participant exclaimed:

I now have the passion to go and help pupils who are in dire need of sexuality HIV and AIDS knowledge*. (Edith, 56-year old teacher)*

After some further reflection, Edith, demonstrates eagerness and motivation to go forward and teach sexuality. Likewise, another participant also added:

What has changed in me, about my future teaching is to continue teaching pupils despite different challenges coming by cultural differences. *(Rose, 51- year old teacher)*

An important learning for the participants was that they could not force learners to comply with what they taught. The teachers could only enable the learners to make informed decisions.

Another participant shared her experience as she declared:

I teach all those factors in G&C lessons, and the pupil have to decide which path to follow. She went on to say, I shall teach, and they shall make choices in life. *(Sarah, 52-year old teacher)*

In agreement, another participant also expressed herself:

Now, I know, I used to be biased, saying, do as I say, but now I shall teach, so that they can make their own choice. *(Nyasha, 36 years old teacher)*

It is difficult not to show your own biases when teaching sexuality education, and it was important for the participants to come to the realisation that they should teach the curriculum in an unbiased way, however difficult it might be, and not preach what they believed in, and assume that they are the only ones who know it all. Literature concurs that teachers should guard against bias and be open to engage with existing issues in sexuality education (Pattman & Chege, 2003; Visser, 2004), and not reinforce their normative thinking as the only truth (Francis, 2012a; Theron, 2007; Wood, 2009b). The participants in this study did show evidence of an awareness of how personal biases might affect their teaching. For instance, Sarah said that she was going to teach the curriculum as it is without ‘editing’, and it would be up to the learners to make their own choices.

#### 6.3.4. Seeing possibilities for a new participatory method of teaching

The teachers experienced a new research method, drawing, and seemed to be enthusiastic about applying it as a participatory pedagogy in their own classes – enabling the learners to participate in talking about crucial and sensitive issues. One participant had this to say:

I see a bright future in the teaching of sexuality education as my children shall use drawing. *(Esinia, 38-year old teacher)*

Furthermore, another participant also echoed:

In my future teaching, I would ask the pupils to be open by using drawings to explain themselves*. (Vongai, 42-year old teacher)*

One participant in addition, acknowledged that:

There are so many methods to use, I have learnt one new way, which is drawing. *(Nyasha, 36 years old teacher)*

In perceiving the possibility of using a new participatory method, another participant also remarked that:

I have learnt that use of drawing is very effective in explaining crucial issues. *(Esther, 56-year old teacher)*

Using drawing seemed to open up the possibility of participants using other methods too in a participatory way, such as collage, paving the way for a learner-centred approach. Another participant responded as follows:

Even those pamphlets can be used to impart knowledge as children cut pictures and discuss*. (Sarah, 52-year old teacher)*

In concurrence, another participant articulated:

Leaflets, fliers and posters are there, we go and collect them and use them in class. If you go to family planning, you get these charts, you can ask your children to cut the pictures, they will like it. There are posters which children can cut and discuss, issues like stigma and discrimination. *(Rose, 51- year old teacher)*

While a learner centred approach and using participatory visual methods are suggested, the teachers also realised that the reality of a heavy workload and teaching large classes may hamper participation, as one participant expressed:

I have 36 lessons and 52 children in one class, yes, I may like to use them in groups, but it is not possible, my classes are too big, the classroom is full, no space. *(Sarah, 52-year old teacher)*

In addition, another participant also argued:

It depends with the load; you can always use these participatory methods. *(Edith, 56-year old teacher)*

Participatory methods involve learners learning individually or in groups doing different types of activities. In this study participants referred to smaller classes as more conducive to using participatory methods. This is consistent with the argument put across by Liew (2014), who asserts that pupil discomfort in sexuality education lessons is high, often manifesting itself in reluctance to participate constructively because of class sizes and composition for instance in terms of sex, ability, maturity and age. Choice of materials to use during participatory class activities also determines the level of participation (Liew, 2014), in this instance, engaging with a sensitive topic. Drawing is therefore seen as a powerful tool to use in teaching since it can generate discussion around an issue of interest amongst participants (Stuart, 2007). When teachers employ this method in their lessons, learners become active participants (Theron, 2007) and with such type of learning, valid co-production of knowledge occurs.

#### 6.3.5. Together becoming ‘overcomers’

A central aspect of participatory visual research is that the participants not only co-produced knowledge, but they learnt from each other. The G&C teachers seemed to learn about sexuality education from each other but also collaboratively found solutions to the challenges they identified. One of the participants had the following experience to share:

I learnt a lot about challenges to teaching sexuality education, and some solutions*. (Edith, 56-year old teacher)*

Additionally, another participant also uttered:

My view of teaching the subject has improved having gained information from other teachers*. (Rose, 51- year old teacher)*

Likewise, there was yet another comment from another participant:

I learnt that there are some challenges in teaching sexuality education in G&C, however, there are some possible solutions to all these challenges. *(Sarah, 52-year old teacher)*

Another participant also expressed herself saying:

These points will make me an effective G&C teacher or counselor*. (Vongai, 42-year old teacher)*

Similar sentiments were also echoed by yet another participant who exclaimed:

We looked at how to overcome the challenge that teachers are meeting, now we are overcomers. *(Shuvai, 51-year old teacher)*

If teachers are to overcome most classroom challenges, building good relations with learners is key (Berglas et al., 2014). Working together as a team is beneficial in overcoming the challenges experienced (Helleve et al., 2011). Additionally, it is from the participatory visual methodology process of this study, and the space created by the research which allowed for comfortable conversation, that G&C teachers critically thought of how they could assist each other to teach the critical content in sexuality education and also how they were enabled in some ways. There is evidence of thoughtful analysis and reflection by the participants, which according to Willis (2007) is typical of the critical paradigm.

## 7. Discussion

**Figure 1.**
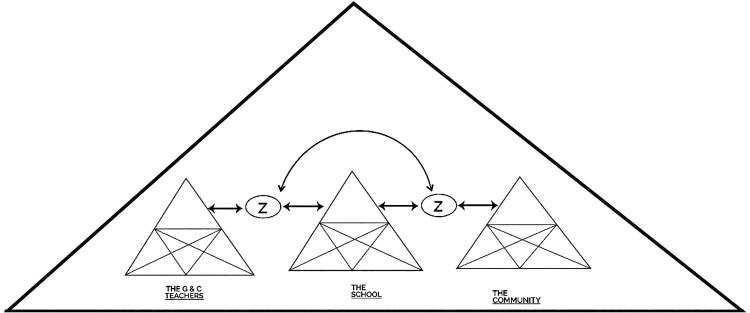
The three Activity Systems to function as one activity system.

Having discussed the findings and recontextualising it in literature, we return to the theoretical framework, Cultural Historical Activity Theory (CHAT), in particular the third generation attributed to Engeström (2001), and posit that in the instance of this study, there are several Activity Systems which effect the shared goal (Z in Figure 1) of enabling G&C teachers to teach the critical sexuality education content to secondary school learners. These three Activity Systems (the collective of G&C teachers, an Activity System of the school, and the Activity System of the community) should be interacting and working in association with each other to enable G&C teachers to teach sexuality education within the HIV and AIDS education curriculum suitable for Zimbabwe secondary school context (See Figure 1).

The Activity Systems we refer to, comprise of subjects (the G&C female teachers), the school community (other teachers, heads of school, education officers, school development committees, non-teaching staff, learners and other actors), and the broader community (parents, learners, and local community leadership). For the participants - the G&C teachers - to achieve their outcome, they have to draw on mediating artefacts or tools which as far as the study is concerned, are the conceptual and material resources needed to teach sexuality education. They have to draw on rules which inform their activity, which in this study are the class regulations, Ministry’s Circulars, school regulations, and the community values and beliefs. They also have to draw on the curriculum which contains philosophical underpinnings, grade specific content, and the suggested methodology. The activity happens within a community in which members achieve their object(ive) through the division of labour. In this study the division of labour refers to the G&C teachers who teach, the school heads who supervise the G&C teachers and who also generate the timetables, and the Education Officers who are employed to supervise G&C teaching. History and culture are important, in that every participant in an Activity System has her own history or lived experiences as a result of her specific cultural background. Therefore, the Activity System is comprised of many voices (Engeström, 2006), in this study, the voices of the eight female participants who each have their own viewpoints, traditions and practices. Consequently, according to CHAT, if all the Activity Systems referred to interact as explained, the G&C teachers could be enabled to teach the critical content in the sexuality education within the HIV and AIDS education curriculum, which is the object and or outcome of the study.

The three Activity Systems each have subjects (members) who dialogue about sexuality education, bringing to the fore contradictions and tensions, which according to CHAT, are the key attributes with the potential to bring about social transformation, change and development in the participants (Engeström, 2001, 2006). In this study where G&C secondary school teachers formed an Activity System, the dialogue around teaching sexuality education, who should teach sexuality education, the apparent lack of mediating artefacts or tools, how participants should talk about sexuality in class in the vernacular or English, the tensions between the curriculum, the teachers’ values, and the teachers or the learners’ cultures seemed to reveal some contradictions. These contradictions and tensions, through the deep discussion enabled a shift in the G&C teachers’ thinking and, hence, possibly some transformation.

The robust discussion which produced dialectics and ambiguities are conceptual tools which emanate from the interaction of the subjects (the G&C teachers) and the other Activity Systems, and which enabled reflexivity. Reflexivity, according to Engeström (2008), is also a mediating tool with participants themselves acting as sources of knowledge on how they can enable themselves to teach sexuality education within HIV and AIDS education curriculum within the Zimbabwe secondary school situation. They mediated their knowledge first among themselves learning from each other - also reflecting on their own identity and its interface with teaching sexuality education - during engagement with a participatory visual method and were then seemingly enabled to mediate the sexuality education knowledge to the learners, who again could mediate it to the communities in order to prevent the spread of HIV infections.

In this article, we therefore have argued that in the instance of enabling G&C teachers to teach sexuality education, three interacting Activity Systems operating as one Activity System is needed to achieve the objective of the study. The G&C teachers were teaching in a school which demonstrated two Activity Systems, the G&C teachers and the school. These, however, require interaction with a third Activity System - the custodian of culture - the wider community. As such, we argue that the wider community is a significant and influential third Activity System, which is often mentioned, but not engaged with in terms of culture. The wider community is where teachers and the learners live and comprises of a multiplicity of interrelated Activity Systems that are interlinked and affect and influence the teaching of sexuality education in the school to learners from diverse cultures. This is where all the culture, the history that CHAT refers to, is embedded. The three Activity Systems i.e. the G&C teachers, the school, and the community, should therefore act as one system, with the subjects, artefacts, communities, rules, division of labour of all three Activity Systems working generatively as one Activity System, towards the achieving of the same objective.

## 8. Conclusion

Returning to the research question of how G&C teachers can be enabled to teach the necessary critical content in sexuality education in the HIV and AIDS education curriculum in Zimbabwe secondary schools, we conclude that the participatory visual methodology used in this study enabled teachers to critically reflect on themselves and their own positioning and its influence on their work, learn from each other, reflect on how the community context could become a space for talking about sexuality; how the school context could become a space to teach sexuality education in light of the diverse cultural backgrounds of the learners, and how they themselves could be more aware of their own cultural positioning in relation to teaching sexuality education. It seemed that they viewed the synchronisation or collaboration of the community, the school and themselves as key in enabling them to transform their realities and enable their agency to teach the critical sexuality education content in their secondary schools in Zimbabwe.

The study has several implications for policy in terms of the Guidance and Counseling curriculum, in that the curriculum should enable and encourage a criticality which could open up dialogue about sexuality education and cultural issues in the context of HIV and AIDS. It also has implications for practice in terms of teacher professional development and teacher training in that the such work should be in depth, should enable reflexivity, encourage looking at the teacher’s own positioning, as well as the use of participatory methods which not only draw on the learners’ knowledge and experiences they bring to class but also ease discussion. The study also implies collaboration among all stakeholders to ensure the best possible sexuality education for learners in the context of HIV and AIDS.
